# Circulating metabolite signatures mediating the association between minerals in domestic water and incident MASLD: a prospective cohort study

**DOI:** 10.3389/fnut.2026.1866771

**Published:** 2026-08-26

**Authors:** Keyang Li, Yincheng Liu, Wenlong Li, Li Qin, Danrui Zhang, Hong Chen, Zhiyu Yin, Hanwen Zhou, Biao Xie, Xiaoni Zhong

**Affiliations:** School of Public Health, Chongqing Medical University, Chongqing, China

**Keywords:** alanine, epidemiology, glutamine, lactate, MASLD, minerals

## Abstract

**Background:**

Epidemiological evidence on minerals in domestic water and incident metabolic dysfunction-associated steatotic liver disease (MASLD) remains limited, particularly regarding potential mediating roles of circulating metabolites. We evaluated the association between minerals and incident MASLD and characterized metabolomic biomarkers that may mediate this association.

**Methods:**

We included 218,339 UK Biobank participants without MASLD or other liver disease at baseline. Domestic water hardness (DWH), including calcium (Ca), magnesium (Mg), and calcium carbonate (CaCO_3_), was obtained from water-utility outputs. MASLD was ascertained using linked health records. One hundred sixty-eight plasma biomarkers were measured using nuclear magnetic resonance profiling. We used Cox models to quantify associations between minerals and MASLD risk, while causal mediation analyses were used to evaluate mediation by these biomarkers.

**Results:**

During a median follow-up of 13.7 years, 3,004 participants developed MASLD. Higher Ca in domestic water was positively associated with incident MASLD (Hazard ratio 1.087, 95% confidence interval 1.013–1.166), whereas Mg (1.042, 0.993–1.094) and CaCO_3_ (1.006, 0.939–1.078) showed no significant associations with MASLD. In mediation analyses, Glutamine, Lactate, and Alanine may mediated the association, with proportions mediated of 21.42, 7.53, and 5.38%, respectively.

**Conclusion:**

Ca in domestic water was positively associated with incident MASLD, with partial mediation by metabolites including glutamine, lactate, and alanine. These findings provide insight into the metabolic pathways of minerals-MASLD association and support prevention strategies.

## 1 Introduction

Metabolic dysfunction-associated steatotic liver disease (MASLD), previously referred to as non-alcoholic fatty liver disease (NAFLD), is characterized by diffuse hepatocellular lipid accumulation and dysregulated lipid metabolism (1). It has emerged as an important driver of a wide range of diseases, including cardiovascular disease, type 2 diabetes, and chronic kidney disease, and currently affects an estimated 38% of the global adult population (2, 3). Although the associated disease burden is substantial, it can be significantly reduced by targeting metabolic disorders through interventions on modifiable factors.

Domestic water mineral composition, commonly reflected by domestic water hardness (DWH), includes the concentration of calcium (Ca), magnesium (Mg), and calcium carbonate (CaCO₃). While DWH has been associated with the development and progression of atopic eczema, psoriasis, cardiovascular diseases, and bladder cancer (4–7), its impact on MASLD remains unclear. Further, previous studies of Ca and Mg have largely focused on intake from diet and dietary supplements. In contrast, studies of DWH consider minerals as an environmental exposure, reflecting chronic, low-dose intake that differs from dietary sources in chemical form, bioavailability, and consumption patterns. These characteristics suggest that DWH may engage metabolic pathways, which could underlie its potential impact on MASLD risk incidence.

Although the biological mechanisms linking DWH to MASLD remain limited, existing evidence points to several plausible metabolic pathways. Studies have shown that Ca and Mg from drinking water are absorbed into the bloodstream within 2–4 h after ingestion (8, 9). Beyond serving as intracellular second messengers, Ca and Mg are essential for the activity of multiple hepatic metabolic enzymes. For instance, serum Mg deficiency, frequently observed in patients with type 2 diabetes, has been associated with higher concentrations of circulating free fatty acids and triglycerides, along with reduced high-density lipoprotein (HDL) concentrations (10, 11), all of which represent early biomarkers of MASLD. These observations suggest that disturbances in metabolic pathways involving Ca and Mg homeostasis may contribute to the development of MASLD. These pathways can be further characterized at metabolomic concentrations using NMR-based profiling in combination with mediation analysis. However, epidemiologic evidence integrating DWH, metabolomic signatures, and MASLD risk remains limited. In this context, NMR-based metabolomic profiling with mediation analysis provides a useful framework to better characterize the relationship between DWH and MASLD by quantifying the mediating role of metabolomic features in this association, thereby providing epidemiologic evidence on the potential metabolic pathways linking DWH to MASLD.

To address this knowledge gap, we conducted a prospective cohort study within the UK Biobank to systematically evaluate the association between DWH and the risk of MASLD. Furthermore, we applied mediation analysis to explore the potential roles of circulating metabolites in mediating the effects on MASLD development. This study aims to provide novel prospective evidence on environmental exposures and MASLD pathogenesis, and to inform strategies for early and targeted prevention.

## 2 Methods

### 2.1 Study population and design

The UK Biobank is a large, population-based prospective cohort that recruited more than 500,000 adults (12). At baseline, participants attended an assessment centre where they completed a touchscreen questionnaire and a verbal interview to collect information on sociodemographic characteristics, lifestyle factors, and medical history. A range of physical measurements was performed, along with the collection of blood, urine, and saliva samples. A total of 502,139 participants were initially included, we excluded participants (1) with prevalent MASLD (*n* = 462) or any other liver disease at the baseline survey (13) (*n* = 9,700) (ICD codes available in Supplementary Table S1); (2) missing data on Ca and Mg (*n* = 46,036); (3) without available NMR metabolomic data (*n* = 209,229); (4) with missing information on covariates (*n* = 18,373). Finally, 218,339 participants were included in the analysis. A flowchart illustrating the study design and participant selection is presented in Supplementary Figure S1.

### 2.2 Ethics approval and consent to participate

This study used the UK Biobank Resource (application number 160957). The UK Biobank study received ethical approval from the Northwest Multi-Centre Research Ethics Committee. All participants provided written informed consent, and the study was conducted following the STROBE guidelines for reporting observational epidemiology (Ref: 21/NW/0157, https://www.ukbiobank.ac.uk/about-us/how-we-work/ethics/).

### 2.3 Outcome

The diagnosis of MASLD was ascertained using ICD-10 codes K76.0 and K75. 8. Follow-up time was from each participant’s baseline assessment date to the earliest occurrence of MASLD diagnosis, death, censoring due to loss to follow-up, or the administrative end of follow-up. Health data were available up to 31 October 2022 for England, 31 August 2022 for Scotland, and 31 May 2022 for Wales.^1^

### 2.4 Assessment of domestic water hardness

Data were obtained from the UK Biobank (Category 603). Residential postcodes of participants were linked to water quality monitoring reports provided by regional water supply companies. For each participant, annual mean concentrations for 2005, the year preceding baseline recruitment, were used to represent residential exposure (14). Ca and Mg concentrations were expressed in milligrams per liter (mg/L), and CaCO₃ concentrations were derived from total hardness data reported by the water companies.

### 2.5 Assessment of circulating NMR metabolic biomarkers

NMR metabolic biomarkers were quantified in EDTA plasma samples from UK Biobank participants using a high-throughput metabolomics platform developed by Nightingale Health Ltd. (Helsinki, Finland) (15). For this study, we used data on 168 biomarkers measured as absolute concentrations between 2019 and 2022. These biomarkers cover multiple metabolic pathways, including lipoprotein lipids in 14 subclasses, fatty acids and their relative compositions, and a range of low-molecular-weight metabolites such as amino acids, ketone bodies, and glycolysis-related metabolites. The protocol has been described on the website: https://biobank.ctsu.ox.ac.uk/crystal/label.cgi?id=220.

### 2.6 Covariates

Based on previous studies (7, 16), a directed acyclic graph was developed to select the minimum sufficient adjustment set (Supplementary Figure S2). Covariates assessed at baseline were considered as potential confounders. Socioeconomic characteristics included age (years), sex (female or male), assessment centre (England, Scotland, or Wales), ethnicity (White or non-White), education (college or above, high school or less than high school), and the Townsend deprivation index (deprivation score derived from residential postcode). Lifestyle factors included BMI (kg/m^2^, calculated as weight in kilograms divided by height in metres squared and modelled as a continuous variable), smoking status (never, former or current), alcohol consumption (non-drinker, <1 g/day, 1–10 g/day, 10–20 g/day or ≥20 g/day), sleep duration (hours per night), healthy diet index [HDI, continuous scores from 0 to 5 calculated based on the intake of vegetables, fruits, fish, processed meat, and unprocessed red meat (17)] and physical activity (sufficient or insufficient). Medication history included medication for metabolic disease (lipid-lowering, antihypertensive, and glucose-lowering drugs, exogenous hormone therapy in women) and use of dietary supplements (Yes or No). Detailed definitions and coding of all covariates are provided in the Supplementary material (covariates).

### 2.7 Statistical analysis

Baseline characteristics were summarized as means with standard deviations (SDs) for continuous variables and as frequencies (proportions) for categorical variables. Metabolites were log-transformed [log(*x* + 1)]. Cox proportional hazards models were used to estimate associations between DWH and risk for incident MASLD. Model 1 was the crude model without any adjustment. Model 2 was adjusted for age, sex, centre, ethnicity, and BMI. Model 3 was additionally adjusted for education, TDI, alcohol intake, HDI, sleep duration, supplement use, and medication for metabolic diseases. Results were reported as hazard ratio (HR) with 95% confidence intervals (CI) for each interquartile range (IQR) increase in DWH. The proportional hazards assumption was evaluated using Schoenfeld residuals and was not violated. Restricted cubic splines with four knots were used to assess the exposure-response association between DWH and MASLD risk. Further, we conducted subgroup analyses to assess potential effect modification by age, sex, BMI, smoking status, alcohol consumption, physical activity, and medication use. The details of subgroups are described in the Supplementary material. Heterogeneity across subgroups was evaluated using a chi-square (χ^2^) test. Sensitivity analyses included the following: (1) to assess residual confounding related to residential location and urban co-exposures, we additionally adjusted for PM2.5, traffic noise, greenness, and urban–rural status, (2) adjust for baseline survey year to reduce potential time-related unmeasured confounding, (3) further adjust for water intake to account for variation in individual-concentrations exposure, (4) include participants without protein measurements and evaluate the consistency of the results, (5) exclude participants who developed the outcome within the first 2 years of follow-up to examine potential reverse causation, (6) exclude participants with less than 10 years of residence to reduce potential exposure misclassification due to recent relocation, (7) calculated the E-value for the primary association between drinking-water Ca concentration and incident MASLD to assess the potential influence of unmeasured confounding. Multivariable linear regression was used to assess associations between DWH and metabolomic biomarkers, and Cox proportional hazards models were used to evaluate associations between metabolites and incident MASLD. To investigate potential mediating pathways, we evaluated 168 circulating metabolites by mediation analysis. Mediation analyses were conducted using the “CMAverse” package in R, and models were adjusted for the same set of covariates, and 95% CI were estimated using 200 bootstrap replications.

All statistical analyses were conducted using R software (version 4.4.0). A two-sided *p-*value < 0.05 was considered statistically significant unless otherwise specified. To account for multiple testing, the Benjamini-Hochberg procedure was applied separately within each of the following analyses: the subgroup analyses (13 grouping variables), the association analyses of 168 circulating metabolites with Ca, the association analyses of 168 circulating metabolites with incident MASLD, and the mediation analyses. An FDR-adjusted *p-*value < 0.05 was considered statistically significant for these analyses.

## 3 Result

### 3.1 Baseline characteristics of study participants

Baseline characteristics of the study population are presented in Table 1. A total of 218,339 participants were included in the analysis. The mean age was 56.47 ± 8.07 years, and 54.1% were female. The median follow-up was 13.7 years, during which 3,004 incident cases of MASLD were identified. Baseline demographic characteristics are summarized in Table 1. Approximately half of the participants reported using dietary supplements, and nearly one-third were taking medications for metabolic conditions. The distributions of DWH and its mineral components are shown in Supplementary Table S2. The mean (SD) concentrations of Ca, Mg, and CaCO_3_ in DWH were 45.48 mg/L, 4.43 mg/L, and 120.04 mg/L, respectively.

**Table 1:** Baseline characteristics of the study participants.

Variables	Total	MASLD	Non-MASLD
	*N* = 218,339	*N* = 3,004	*N* = 215,335
Demographic characteristics			
Age, years	56.47 ± 8.07	56.85 ± 7.86	56.47 ± 8.08
Gender (%)			
Female	118,056 (54.1)	1,572 (52.3)	116,484 (54.1)
Male	100,283 (45.9)	1,432 (47.7)	98,851 (45.9)
Centre (%)			
England	197,361 (90.4)	2,864 (95.3)	194,497 (90.3)
Scotland	13,472 (6.2)	76 (2.5)	13,396 (6.2)
Wales	7,506(3.4)	64 (2.1)	7,442 (3.5)
Race (%)			
White	209,352 (95.9)	2,850 (94.9)	206,502 (95.9)
Non-White	8,987 (4.1)	154 (5.1)	8,833 (4.1)
Education (%)			
College or above	72,535 (33.2)	697 (23.2)	71,838 (33.4)
High school	110,434 (50.6)	1,613 (53.7)	108,821 (50.5)
Less than high school	35,370 (16.2)	694 (23.1)	34,676 (16.1)
Townsend deprivation index	−1.46 ± 3.00	−0.58 ± 3.35	−1.47 ± 2.99
Body mass index, kg/m^2^	27.35 ± 4.72	31.13 ± 5.59	27.29 ± 4.68
Lifestyle factors			
Smoke (%)			
Never	120,780 (55.3)	1,378 (45.9)	119,402 (55.4)
Current	21,340 (9.8)	418 (13.9)	20,922 (9.7)
Previous	76,219 (34.9)	1,208 (40.2)	75,011 (34.8)
Alcohol intake (%)			
<1 g/day	40,251 (18.4)	738 (24.6)	39,513 (18.3)
1–10 g/day	53,355 (24.4)	594 (19.8)	52,761 (24.5)
10–20 g/day	48,679 (22.3)	476 (15.8)	48,203 (22.4)
≥20 g/day	60,619 (27.8)	844 (28.1)	59,775 (27.8)
Non-drinker	15,435 (7.1)	352 (11.7)	15,083 (7.0)
Physical activity (%)			
Sufficient	165,153 (75.6)	2053 (68.3)	163,100 (75.7)
Insufficient	53,186 (24.4)	951 (31.7)	52,235 (24.3)
Health dietary index	3.66 ± 1.52	3.41 ± 1.56	3.66 ± 1.52
Duration of sleep (hours)	7.13 ± 1.19	7.04 ± 1.51	7.14 ± 1.18
Medication history			
Supplements use (%)			
Yes	110,878 (50.8)	1,478 (49.2)	109,400 (50.8)
No	107,461 (49.2)	1,526 (50.8)	105,935 (49.2)
Medication for metabolic disease (%)			
Yes	68,818 (31.5)	1,485 (49.4)	67,333 (31.3)
No	149,521 (68.5)	1,519 (50.6)	148,002 (68.7)

Continuous variables are presented as the mean ± SD. Categorical variables are presented as numbers (percentages). MASLD, metabolic dysfunction-associated steatotic liver disease.

### 3.2 Associations between DWH exposure and incident MASLD risk

**Figure 1:**
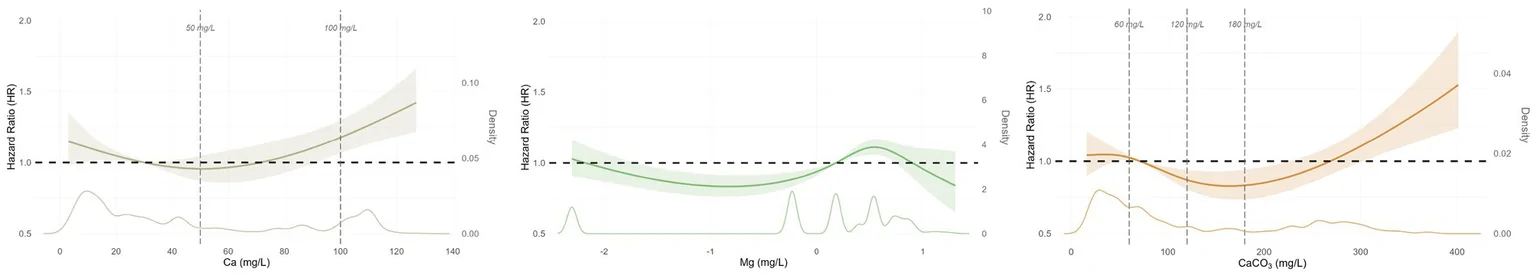
Restricted cubic spline analyses of the associations between domestic water Ca, Mg, and CaCO_3_ concentrations and incident MASLD.

The associations between exposure and the risk of MASLD are presented in Table 2. In Model 1, higher Ca concentrations were positively associated with MASLD risk (95% CI:1.061–1.206), and this association remained significant after full adjustment for potential confounders (1.013–1.166). Mg showed a positive association in the crude model (1.064–1.157), but this relationship was attenuated and became non-significant after adjustment (0.993–1.094). CaCO_3_ was not significantly associated with MASLD risk across any of the models. These findings suggest a potential role of Ca in the development of MASLD, whereas the associations with Mg and CaCO_3_ remain inconclusive. In Figure 1, the results of RCS provided evidence of non-linear exposure-response associations (Ca: *P*-nonlinear < 0.001; Mg: *P*-nonlinear < 0.001; CaCO_3_: *P*-nonlinear < 0.001). Visually, the curves for Ca and CaCO_3_ showed a U-shaped pattern, with relatively lower MASLD risk at intermediate concentrations and higher risk at both lower and higher concentrations. As shown in Supplementary Figure S3, serum Ca concentrations showed a gradual increase with higher Ca levels in domestic water. The subgroup results are shown in Supplementary Table S3. The association between Ca and incident MALSD risk appeared stronger among participants who had higher alcohol intake, were obese, and were of non-White ethnicity, indicating a higher risk in these subgroups. In the sensitivity analyses, the associations were not materially changed (Supplementary Table S4).

**Table 2:** Associations of DWH and incident MASLD risk.

Exposure	Model 1		Model 2		Model 3	
	HR (95% CI)	*p*	HR (95% CI)	*p*	HR (95% CI)	*p*
Ca	1.131 (1.061, 1.206)	<0.01	1.080 (1.010, 1.154)	0.02	1.087 (1.013, 1.166)	0.01
Mg	1.109 (1.064, 1.157)	<0.01	0.996 (0.982, 1.076)	0.22	1.042 (0.993, 1.094)	0.08
CaCO_3_	1.019 (0.957, 1.085)	0.53	0.986 (0.924, 1.052)	0.68	1.006 (0.939, 1.078)	0.84

Ca, calcium; Mg, magnesium; CaCO_3_, calcium carbonate; HR, hazard ratio; CI, confidence interval.

Model 1 was the crude model without any adjustment. Model 2 was adjusted for age, sex, centre, ethnicity, and BMI. Model 3 was additionally adjusted for education, TDI, alcohol intake, HDI, sleep duration, supplement use, and medication for metabolic diseases. HR was estimated per IQR increase in the corresponding drinking-water mineral.

### 3.3 Potential mediating role of metabolic biomarkers

**Figure 2:**
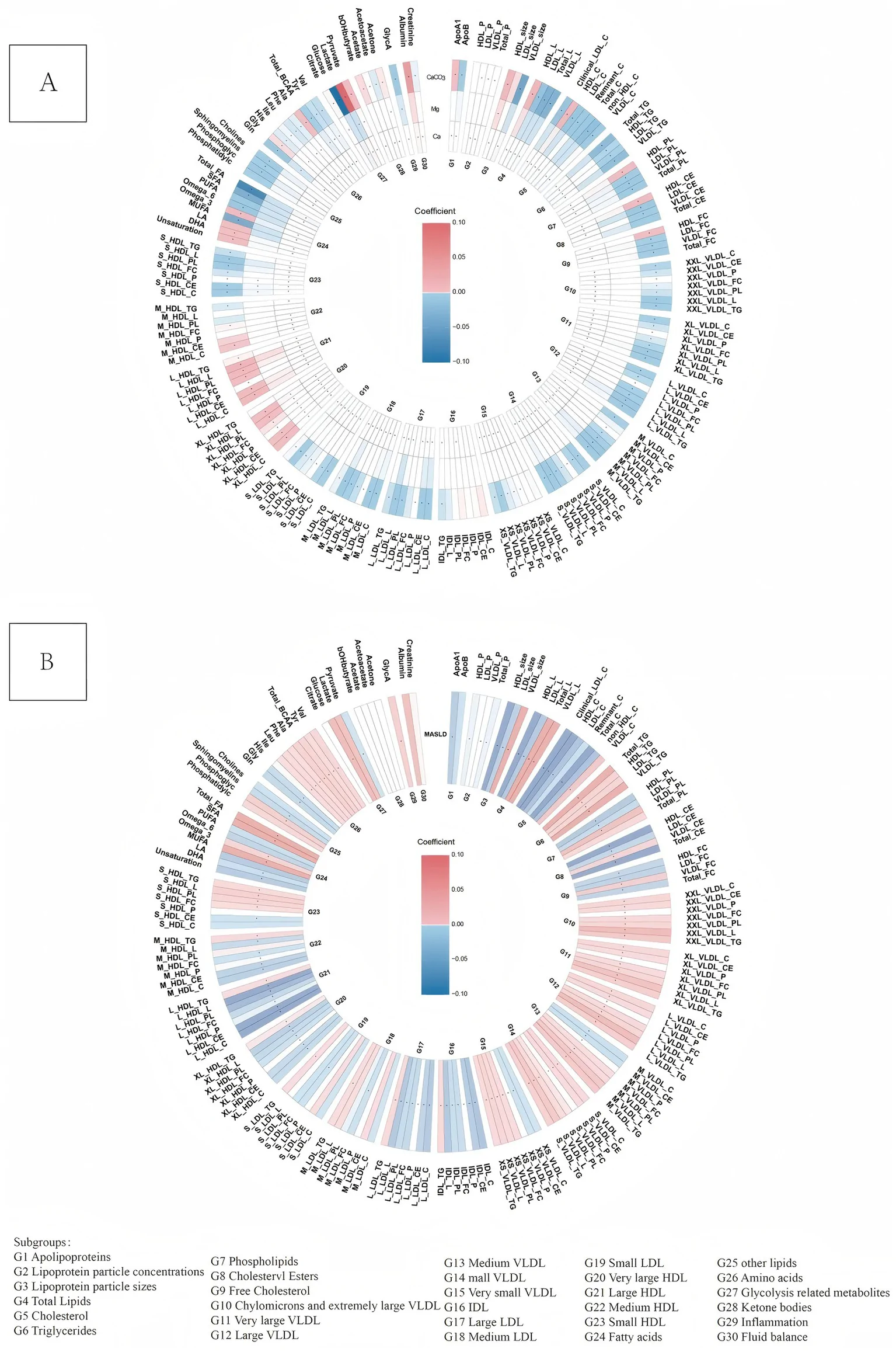
Associations of 168 circulating metabolites with Ca and incident MASLD. **(A)** Associations between Ca and 168 circulating metabolites. **(B)** Associations between 168 circulating metabolites and incident MASLD. Metabolites are grouped into 30 predefined subclasses (G1–G30). Colors represent the direction and magnitude of the regression coefficients, with red indicating positive associations and blue indicating negative associations. The Benjamini-Hochberg procedure was applied separately within each analysis, and an FDR-adjusted *p-*value < 0.05 was considered statistically significant.

**Figure 3:**
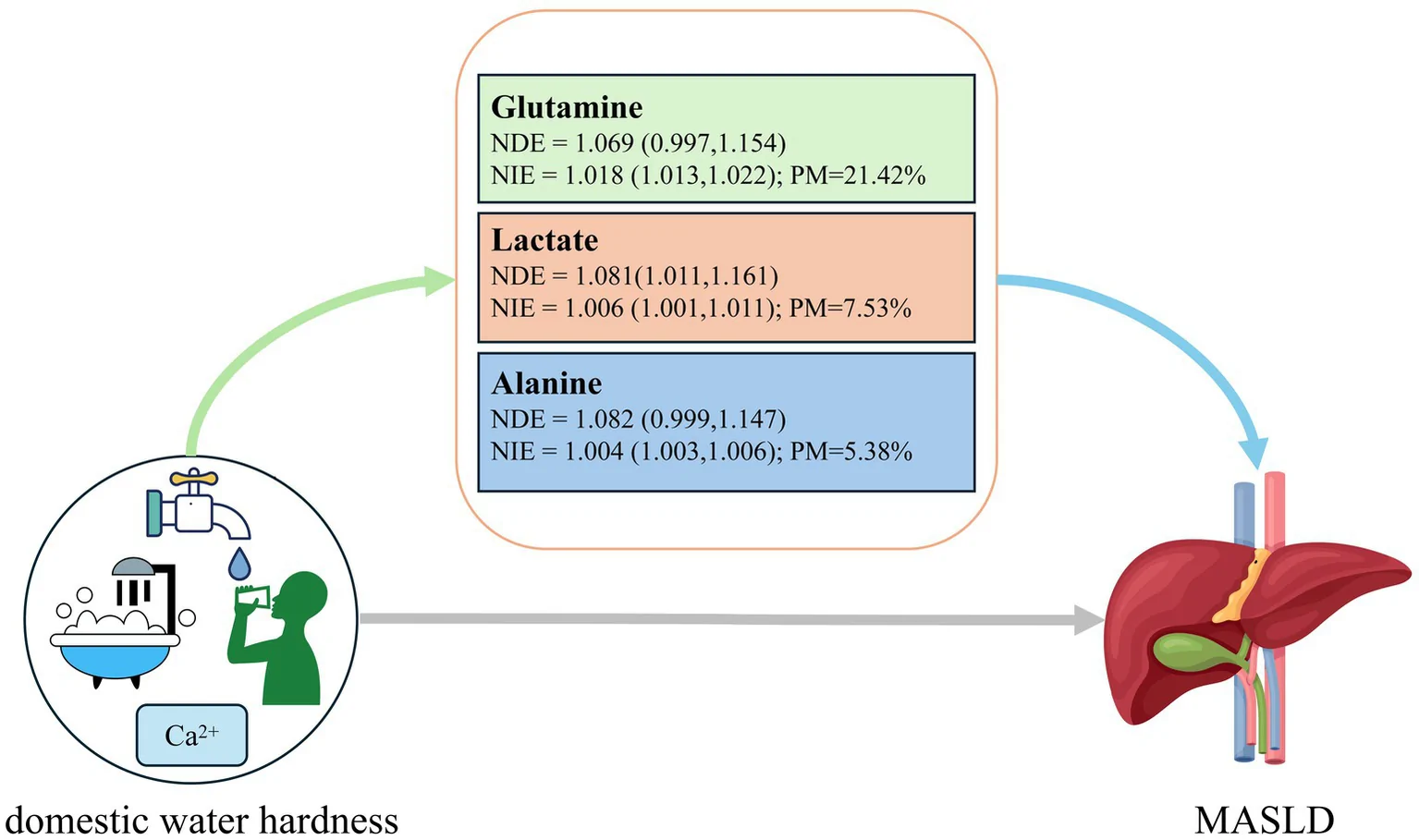
Mediation analysis with circulating metabolites as potential mediators for the associations between Ca and incident MASLD risk. Glutamine, lactate, and alanine were identified as key circulating metabolites mediating the association between drinking water hardness and incident MASLD. The values shown are the mediation effect estimates with 95% confidence intervals and corresponding mediation proportions. NIE, the natural indirect effects; NDE, the natural direct effects; PM, proportion mediated.

First, we assessed the associations of each of the 168 circulating metabolites with Ca and with incident MASLD, with the FDR controlled at *p* < 0.05 within each analysis, as shown in Figure 2. To further explore the potential role of circulating metabolites in the pathway from DWH to MASLD, we conducted mediation analyses for all 168 metabolites and presented the three metabolites with a proportion mediated greater than 5% (Figure 3). The natural indirect effects (NIE) of Ca on MASLD risk were significant for all three metabolites, whereas the natural direct effects (NDE) were significant only for lactate. Specifically, for glutamine, HR__NIE_ = 1.018 (95% CI 1.013–1.022, *p* < 0.001) and HR__NDE_ = 1.069 (95% CI 0.997–1.154, *p* = 0.08), with 21.42% of the total effect mediated; for lactate, HR__NIE_ = 1.006 (95% CI 1.001–1.011, *p* = 0.03) and HR__NDE_ = 1.081 (95% CI 1.011–1.161, *p* = 0.04), with 7.53% mediated; and for alanine, HR__NIE_ = 1.004 (95% CI 1.003–1.006, *p* < 0.001) and HR__NDE_ = 1.082 (95% CI 0.999–1.147, *p* = 0.07), with 5.38% mediated. The mediation results for the remaining metabolites are provided in Supplementary Table S6.

## 4 Discussion

In this large prospective epidemiological cohort study, we systematically evaluated the association between DWH and the risk of incident MASLD. Additionally, 168 circulating metabolites were used to investigate their potential role in the pathway from DWH to MASLD. To our knowledge, this is the first study to explore this relationship. Unlike previous studies that did not distinguish lipoprotein composition, size, or concentration, our work provides a more refined subclass-specific characterization, offering a deeper understanding of metabolic alterations associated with DWH exposure. Through mediation analysis, we further found that Ca may mediate the development of MASLD through metabolites such as glutamine, lactate, and alanine. These findings provide new insights into the metabolic consequences of environmental exposures and suggest potential strategies for preventing MASLD.

### 4.1 Compared with other studies

Existing studies have examined DWH in relation to digestive, metabolic, or cardiovascular outcomes. For example, He et al. (16) found an association between CaCO_3_ concentrations in domestic water and digestive system disease. A nationwide Dutch study covering approximately seven million individuals reported that higher magnesium concentrations in drinking water were inversely associated with coronary heart disease mortality, whereas higher Ca concentrations and overall water hardness were positively associated with cardiovascular mortality (18). Another study observed that moderate concentrations of CaCO₃ and elevated Mg were linked to a lower risk of cardiovascular events. Whereas excessive exposure was associated with increased risk of chronic rheumatic heart disease (19). Beyond DWH, several studies have focused on Ca and Mg from the diet. A case–control study involving 196 patients found that a higher dietary intake of Ca and Mg was positively associated with NAFLD (19). Similarly, a large cross-sectional study of 16,592 Korean adults found significant associations between serum Ca and phosphorus concentrations and NAFLD risk (20). However, findings from animal studies appear inconsistent with those from large-scale epidemiological studies. For example, experimental evidence in mice suggests that dietary Ca supplementation can attenuate hepatic steatosis induced by a high-fat diet (21). These discrepancies may be attributable to differences in species, mineral sources, chemical forms, exposure concentrations, and exposure duration, all of which may contribute to divergent findings between experimental and epidemiological studies.

In addition, we identified three circulating metabolites, with glutamine inversely associated with MASLD risk, whereas alanine and lactate were positively associated.

Some previous findings supported our results. For instance, a Mendelian randomization analysis has demonstrated that higher alanine and lower glutamine concentrations are causally associated with an increased risk of MASLD (22). Consistently, large prospective cohort data from the Nurses’ Health Study showed that a high alanine-to-glutamine ratio predicts a greater incidence of type 2 diabetes (23). An experimental study further supports this protective role of glutamine, showing that restoration of glutamine concentrations in obese and diabetic mice markedly alleviated oxidative stress and hepatic lipid accumulation (24).

### 4.2 Potential mechanisms

Ca in DWH may differ from dietary or supplemental Ca in terms of exposure characteristics and potential biological effects. Previous studies of calcium-containing mineral waters suggest that dissolved Ca can be efficiently absorbed and may influence calcium-regulatory responses. Pharmacokinetic studies indicate that Ca from mineral water is readily absorbed, with bioavailability comparable to that from dairy sources, and that even single ingestions can induce measurable changes in parathyroid hormone secretion and bone metabolism (9, 25, 26). This exposure pattern contrasts with intermittent bolus doses from Ca supplements and with dietary Ca intake, which is affected by co-consumed nutrients such as phosphate, oxalate, and fat. Therefore, chronic low-dose exposure to ionic Ca may plausibly influence metabolic regulation through sustained, subtle modulation of calcium homeostasis rather than transient increases in Ca availability. However, because circulating Ca concentrations are tightly regulated through intestinal absorption, renal excretion, bone buffering, vitamin D, and parathyroid hormone, these findings should not be interpreted as evidence that Ca from DWH directly accumulates in the circulation or directly causes hepatic injury.

In our study, higher Ca concentrations in DWH were associated with an increased risk of MASLD, and Ca showed a nonlinear association with MASLD risk. Several studies have examined associations between drinking-water exposures and liver diseases, but the available evidence has largely focused on specific contaminants or water-quality indicators such as disinfection by-products, heavy metals, and microbial contamination (27–30). Although direct evidence linking Ca exposure in DWH with MASLD remains limited, previous studies have reported associations between Ca concentrations and cardiovascular or metabolic outcomes (31, 32). Experimental evidence further suggests that disrupted Ca homeostasis in hepatocytes, particularly involving the endoplasmic reticulum-mitochondria axis, may contribute to mitochondrial dysfunction, oxidative stress, and metabolic abnormalities in the liver (33, 34). These findings provide biological plausibility for a potential relationship between Ca in DWH and MASLD, although the underlying mechanisms remain uncertain. Furthermore, Ca concentrations in DWH may correlate with other physicochemical characteristics of water, such as pH, alkalinity, and other dissolved constituents; we cannot exclude the possibility that unmeasured water-quality factors contributed to the observed association. The lack of detailed information on these parameters limited our ability to further disentangle the independent effect of Ca exposure from other correlated aspects of water chemistry.

The association between Ca in DWH and MASLD may also differ according to underlying metabolic susceptibility. In subgroup analyses, the association appeared stronger among individuals with obesity, higher alcohol intake, and non-White participants. These findings suggest that pre-existing metabolic vulnerability or coexisting hepatic stressors may modify susceptibility to *Ca.* However, the stronger association observed among non-White participants should be interpreted with caution given the limited number of events in this subgroup. These interaction findings were exploratory and require confirmation in larger and more diverse populations.

To further explore potential metabolic pathways linking Ca in DHW with MASLD risk, we conducted mediation analyses using circulating metabolites. We identified glutamine, alanine, and lactate as potential metabolic mediators. These metabolites are involved in biological processes related to nitrogen metabolism, the tricarboxylic acid cycle, the glucose-alanine cycle, and glycolysis (35–37). Previous epidemiological and experimental evidence supports their relevance to MASLD. In the Young Finns cohort study, serum glutamine was inversely associated with fatty liver, whereas higher baseline plasma alanine was positively associated with incident metabolic dysfunction-associated fatty liver disease, suggesting that systemic metabolic alterations may precede disease development (38). A Korean long-echo-time proton magnetic resonance spectroscopy study reported higher alanine and lactate concentrations in individuals with steatohepatitis and suggested that alanine may help distinguish steatohepatitis from simple steatosis (39). In addition, clinical evidence indicates that lactate concentrations are increased in liver diseases (40).

Notably, the proportions mediated by lactate (7.53%) and alanine (5.38%) were modest and should therefore be interpreted cautiously. Nevertheless, these findings provide preliminary evidence that metabolic alterations involving these metabolites may contribute to the association between Ca in DWH and MASLD risk. The identified indirect effects remained significant after FDR correction across all 168 metabolites tested, and the directions of association were broadly consistent with previous epidemiological and experimental evidence. The modest mediation proportions also suggest that individual metabolites capture only limited components of a complex metabolic network. Additional biological pathways not represented in the current biomarker panel, as well as residual confounding, may contribute to the observed association. Therefore, these mediation analyses should be considered exploratory and hypothesis-generating rather than definitive evidence of causal metabolic pathways.

Overall, our findings suggest a potential association between Ca concentrations in DWH and MASLD, possibly linked to alterations in metabolic pathways. However, these metabolites explained only part of the association, and the residual association may reflect other unmeasured mechanisms, including inflammation, oxidative stress, insulin resistance, other metabolic pathways, or residual confounding. These findings should therefore be interpreted as exploratory and require further confirmation.

### 4.3 Implications

Given that current drinking-water quality standards pay relatively limited attention to metabolic health outcomes, our results suggest that drinking-water mineral composition, particularly Ca concentration, may represent an environmental exposure worthy of further investigation in metabolic health research. From a public health perspective, systematic characterization and reporting of mineral composition in drinking water may provide useful contextual information for future epidemiological studies and health risk assessments. However, any policy or clinical recommendations regarding modification of drinking-water mineral content would require confirmation from independent populations, regions with different water-quality profiles, and study designs better suited for causal inference. Finally, because the observed associations between mineral exposures and metabolic outcomes may be influenced by residual confounding, further studies in diverse populations and settings are warranted, with improved exposure assessment and deeper investigation of underlying mechanisms to enhance the robustness and generalizability of these findings.

### 4.4 Strengths and limitations

Our study has several strengths. Conducted in a large prospective cohort with 13.7 years of follow-up, it provides a systematic assessment of the association between DWH and MASLD. Furthermore, by employing comprehensive profiling of 168 circulating NMR metabolites, we reveal a novel link between DWH and the metabolic profiles associated with MASLD for the first time. These findings offer new public health insights for environmental health policies, whereas previous research has been confined to minerals from diet or supplements. In addition, we conducted a series of sensitivity analyses to assess the robustness of our results.

Nevertheless, several limitations should be considered in interpreting our findings. First, the age range of UK Biobank participants (40–73 years of recruitment) may not be fully representative of the onset and progression of MASLD across the entire adult lifespan, particularly in younger adults. Second, the DWH data may not capture changes in water quality during distribution, which could bias the exposure assessment. Third, DWH estimates from 2005 were used as an indicator of long-term exposure, but residential mobility during follow-up may have led to some misclassification of individual exposure. To address this concern, we additionally adjusted for residential duration in sensitivity analyses, and the results were consistent with the main analyses, supporting the robustness of our main conclusions. Fourth, because most UK Biobank participants are of European ancestry and volunteer bias exists, the generalizability of our findings to other populations and ethnic groups is limited. Finally, the causal mediation analyses require assumptions that cannot be fully verified in observational studies, particularly regarding the absence of unmeasured confounding among exposure, metabolic mediators, and MASLD risk, as well as exposure-induced mediator-outcome confounding. Although we adjusted for an extensive set of covariates selected using a directed acyclic graph, residual confounding cannot be completely excluded. Therefore, the mediation findings should be interpreted as exploratory and hypothesis-generating and require confirmation in future studies using designs better suited for causal inference.

## 5 Conclusion

Our study shows that higher Ca concentrations in domestic water are associated with an increased risk of incident MASLD, with glutamine, lactate, and alanine potentially explaining a limited proportion of the observed association in exploratory mediation analyses. These findings provide new insights into the metabolic pathways linking environmental mineral exposure to MASLD and may help inform future prevention strategies.

## Data Availability

The data analyzed in this study is subject to the following licenses/restrictions: The UK Biobank data are subject to access restrictions and cannot be publicly shared by the authors. Data access is available to eligible researchers upon application to UK Biobank and approval for health-related research in the public interest. Requests to access these datasets should be directed to the UK Biobank data can be accessed through an approved application (www.ukbiobank.ac.uk).

## Footnotes

1
https://biobank.ndph.ox.ac.uk/showcase/exinfo.cgi?src=Data_providers_and_dates
