# Supplementary material for: Circulating metabolite signatures mediating the association between minerals in domestic water and incident MASLD: a prospective cohort study
Source: Front Nutr. 2026 Aug 26;13:1866771. doi: 10.3389/fnut.2026.1866771 (PMC13553438; doi:10.3389/fnut.2026.1866771)
Supplement: Supplementary file 1 [file Supplementary_file_1.DOCX]

**Supplementary material for Metabolomic signatures mediating the association between minerals in domestic water and incident MASLD: A prospective cohort study**

**Contents**

[Covariates	3](#_Toc23035)

[Subgroup analysis	5](#_Toc29901)

[Figure S1 Participants inclusion process for the study	6](#_Toc8294)

[Figure S2 Directed acyclic graph for the study	7](#_Toc29603)

[Figure S3 The correlation between serum calcium and calcium in drinking water](#_Toc22846)

[Table S1 ICD codes for other liver disease	9](#_Toc9765)

[Table S2 Summary distributions of annal concentrations of DWH indicator at baseline	10](#_Toc13117)

[Table S3 Subgroup analysis for associations between DWH and incident MALSD risk	11](#_Toc10685)

[Table S4 Sensitivity analysis for associations between DWH and incident MALSD risk	14](#_Toc16719)

[Table S5 Mediation analyses for identified metabolomic biomarkers in associations between Ca and incident MASLD risk	15](#_Toc19480)

# Covariates

Here we provided detailed definitions and UK Biobank field IDs for all covariates used in the manuscript.

Covariates included age (field 21022), sex (field 31), location (field 54), education (field 6138), TDI (field 22189), ethnicity (field 21000), smoking status (field 20116), alcohol intake (category 100051), physical activity (category 10054), sleep duration (field 1160), BMI (field 21001), HDI (category 100052), dietary supplementation (field 6155, 6179), medication for metabolic disease (Category 100045). Further, variables requiring additional detail are described below.

***Alcohol intake***

Alcohol intake was derived from self-reported average weekly/monthly intake of red wine (field 1568, 4407), champagne plus white wine (field 1578, 4418), beer plus cider (field 1588, 4429), spirits (field 1598, 4440), fortified wine (field 1608, 4451), and other alcoholic drinks (field 4462, 5364). We assigned 20 grams of alcohol per serving for a pint of beer and 10 grams of alcohol per serving for all other types of alcohol. For each beverage type, weekly intakes were converted to grams per day by dividing by seven and monthly intakes by dividing by thirty. Values were then summed across beverage types to obtain total grams per day. We categorized average daily alcohol intake into five groups: non-drinkers, <1 g/day, 1-10 g/day, 10-20 g/day, and ≥20 g/day.

***Physical activity***

Physical activity was assessed according to the World Health Organization’s physical activity guidelines (1). Adults should engage in ≥150 minutes of moderate-intensity activity per week or ≥75 minutes of vigorous-intensity activity per week, or an equivalent combination of moderate and vigorous activity. Alternatively, adults may do moderate activity on at least 5 days per week or a single weekly bout of vigorous activity (at least 10 minutes). Based on the self-reported type and duration of physical activity, including walking (field 864, 874), moderate (field 884, 894), and vigorous physical activity (field 904, 914), we calculated a binary measure of activity for each participant.

***HDI***

The HDI was calculated based on the reported frequencies of commonly consumed foods, including vegetables (field 1289, 1299), fruits (field 1309, 1319), fish (field 1329, 1339), processed meats (field 1349), unprocessed red meats (field 1359, 1369, 1379, 1389), grains (field 1438, 1448, 1458, 1468).One serving was operationalized as one heaped tablespoon of cooked vegetable, two heaped tablespoons of raw vegetables, one piece of fresh fruit, or two pieces of dried fruit. Seven components were then scored, with one point awarded for each criterion met: 1) fruits ≥3 servings/day, 2) vegetables: ≥3 servings/day, 3) fish: ≥2 servings/week, 4) processed meats: ≤1 serving/week, 5) unprocessed meats: ≤1.5 servings/week, 6) whole grains: ≥3 servings/day, 7) refined grains: ≤1.5 servings/day. The HDI was defined as the sum of these seven component scores, with higher values indicating a healthier dietary pattern.

***Medications***

Medication covariates included dietary supplementation, medication use for metabolic disease. Dietary supplementation was defined from self-reported use of vitamin and mineral supplements (field 6155) or other dietary supplements (field 6179) and coded as a binary indicator. Medication use for metabolic diseases was determined from self-reported data in the UK Biobank. This was defined as the use of any drug for cholesterol, blood pressure, diabetes (field 6177) and the addition of exogenous hormones for women (field 6153) .

# *Subgroup analysis*

Potential subgroups in this study included age (< 60 years, ≥ 60 years), sex (female, male), TDI (categorized into tertiles), ethnicity (white, other), education (less than high school, high school or equivalent, college or above), smoking status (never, current , Previous ), alcohol intake (< 1 g/day, 1-10 g/day, 10-20g/day, and ≥ 20 g/day), BMI (< 25 kg/m^2^, 25-30 kg/m^2^, ≥ 30 kg/m^2^), physical activity (sufficient or insufficient), HDI (< 3, ≥ 4), sleep duration (< 7 hours, 7-8 hours, ≥ 9 hours), dietary supplementation (yes, no), medication use for metabolic diseases (yes, no).

# Figure S1 Participants inclusion process for the study

# Figure S2 Directed acyclic graph for the study

DWH, domestic water hardness; TDI, Townsend deprivation index; BMI, body mass index; HDI, health dietary index;MASLD,metabolic dysfunction-associated steatotic liver disease .

# Figure S3 The correlation between serum calcium and calcium in drinking water

# Table S1 ICD codes for other liver disease

| Disease | ICD codes |
| --- | --- |
| Hemochromatosis | 'E83.1' |
| Wilson | 'E83.0' |
| Alpha-1-antirypsin deficiency | 'E88.0' |
| Chronic hepatitis, unspecified | 'K73.9', ' K73.2' |
| Budd-Chiari | 'K76.5', 'I82.0 ', 'K76.5 ' |
| AIH, PBC, PSC | 'K83.0', 'K74.3', 'K75.4' |
| secondary or unspecified biliarycirrhosis | 'K74.4', 'K74.5' |
| Viral Hepatitis | 'B16', 'B17', 'B18' ,'B19' |
| codes associated with alcohol use  disorder | 'F10' |
| codes associated with consequence of  alcohol | 'E24.4', 'G62.1', 'I42.6', 'K29.2', 'G31.2', 'G72.1',  'K85.2', 'K86.0', 'T51.0', 'T51.9', 'Y57.1', 'Z50.2',  'Z71.4', 'Z72.1', 'K70', 'X65' |
| Codes associated with drug usedisorders except nicotine/caffeine | 'F11', 'F12', 'F13', 'F14', 'F15', 'F18', 'F19' |

# Table S2 Summary distributions of annal concentrations of DWH indicator at baseline

| Variable | Mean | SD | Min | P_25_ | P_50_ | P_75_ | Max |
| --- | --- | --- | --- | --- | --- | --- | --- |
| Ca, mg/L | 45.48 | 38.21 | 3 | 13 | 30 | 86 | 130 |
| Mg, mg/L | 4.43 | 3.82 | 1 | 2 | 3 | 5 | 36 |
| CaCO_3_, mg/L | 120.04 | 101.99 | 16 | 39 | 71 | 228 | 460 |

Note: P_25_, P_50_, P_75_, denotes 25%, 50%, and 75% percentile separately. DWH, domestic water hardness; CaCO_3_, calcium carbonate; Ca, calcium; Mg, magnesium

# Table S3 Subgroup analysis for associations between DWH and incident MALSD risk

|  | **Ca** | | |  | **Mg** | | |  | **CaCO_3_** | | |
| --- | --- | --- | --- | --- | --- | --- | --- | --- | --- | --- | --- |
|  | **HR (95% CI)** | ***P*** | ***P* for interaction** |  | **HR (95% CI)** | ***P*** | ***P* for interaction** |  | **HR (95% CI)** | ***P*** | ***P* for interaction** |
| **Age** |  |  | 0.746 |  |  |  | 0.575 |  |  |  | 0.578 |
| < 60 years | 1.10 (1.01,1.21) | 0.027 |  |  | 1.01 (0.97,1.05) | 0.588 |  |  | 1.03 (0.94,1.12) | 0.490 |  |
| ≥ 60 years | 1.05 (0.94,1.17) | 0.371 |  |  | 1.02 (0.97,1.07) | 0.396 |  |  | 0.96 (0.86,1.07) | 0.537 |  |
| **Sex** |  |  | 0.288 |  |  |  | 0.207 |  |  |  | 0.610 |
| Female | 1.04 (0.94,1.14) | 0.396 |  |  | 1.03 (0.98,1.07) | 0.190 |  |  | 1.02 (0.92,1.12) | 0.655 |  |
| Male | 1.13 (1.02,1.25) | 0.012 |  |  | 1.00 (0.95,1.04) | 0.944 |  |  | 0.99 (0.89,1.09) | 0.861 |  |
| **TDI** |  |  | 0.127 |  |  |  | 0.909 |  |  |  | 0.238 |
| Q1 | 1.01 (0.91,1.11) | 0.824 |  |  | 1.00 (0.94,1.07) | 0.911 |  |  | 0.93 (0.84,1.03) | 0.184 |  |
| Q2 | 1.00 (0.88,1.13) | 0.938 |  |  | 1.01 (0.95,1.07) | 0.653 |  |  | 0.99 (0.88,1.12) | 0.958 |  |
| Q3 | 1.21 (1.06,1.37) | 0.002 |  |  | 1.02 (0.97,1.07) | 0.320 |  |  | 1.07 (0.95,1.20) | 0.219 |  |
| **Ethnicity** |  |  | **0.020** |  |  |  | **0.020** |  |  |  | 0.028 |
| White | 1.05 (0.98,1.12) | 0.109 |  |  | 1.00 (0.97,1.04) | 0.621 |  |  | 0.98 (0.92,1.05) | 0.690 |  |
| Others | 1.63 (1.12,2.36) | 0.009 |  |  | 1.22 (1.01,1.48) | 0.036 |  |  | 1.37 (0.99,1.91) | 0.057 |  |
| **Education** |  |  | 0.295 |  |  |  | 0.479 |  |  |  | **0.031** |
| Less than high school | 1.06 (0.97,1.17) | 0.165 |  |  | 1.00 (0.93,1.06) | 0.946 |  |  | 1.05 (0.96,1.14) | 0.229 |  |
| High school or equivalent | 1.03 (0.94,1.13) | 0.506 |  |  | 1.00 (0.96,1.04) | 0.894 |  |  | 0.92 (0.84,1.01) | 0.096 |  |
| College or above | 1.21 (1.03,1.43) | 0.020 |  |  | 1.06 (0.99,1.15) | 0.078 |  |  | 1.12 (0.96,1.31) | 0.117 |  |
| **Smoking status** |  |  | 0.978 |  |  |  | 0.09 |  |  |  | 0.839 |
| Never | 1.08 (0.98,1.20) | 0.105 |  |  | 0.98 (0.94,1.03) | 0.649 |  |  | 1.01 (0.91,1.12) | 0.771 |  |
| EVER | 1.08 (0.98,1.19) | 0.092 |  |  | 1.03 (0.99,1.08) | 0.085 |  |  | 0.99 (0.90,1.09) | 0.961 |  |
| **Alcohol intake** |  |  | **0.030** |  |  |  | **0.033** |  |  |  | 0.187 |
| Non-drinkers | 1.19 (0.98,1.45) | 0.069 |  |  | 1.09 (0.98,1.21) | 0.077 |  |  | 1.18 (0.97,1.42) | 0.087 |  |
| < 1 g/day | 1.01 (0.88,1.15) | 0.871 |  |  | 1.05 (0.99,1.12) | 0.086 |  |  | 0.93 (0.83,1.05) | 0.262 |  |
| 1 to 10 g/day | 0.96 (0.82,1.13) | 0.685 |  |  | 0.98 (0.91,1.06) | 0.767 |  |  | 0.99 (0.85,1.14) | 0.900 |  |
| 10 to 20 g/day | 0.97 (0.81,1.17) | 0.814 |  |  | 0.95 (0.87,1.02) | 0.204 |  |  | 0.95 (0.80,1.13) | 0.614 |  |
| ≥ 20 g/day | 1.21 (1.07,1.37) | 0.001 |  |  | 1.00 (0.94,1.06) | 0.918 |  |  | 1.02 (0.91,1.15) | 0.666 |  |
| **BMI** |  |  | **0.005** |  |  |  | 0.143 |  |  |  | **0.001** |
| < 25 kg/m^2^ | 0.95 (0.77,1.18) | 0.694 |  |  | 0.99 (0.89,1.09) | 0.853 |  |  | 0.80 (0.64,1.00) | 0.052 |  |
| 25-30 kg/m^2^ | 1.23 (1.10,1.37) | ＜0.001 |  |  | 1.05 (0.99,1.12) | 0.069 |  |  | **1.14 (1.02,1.26)** | **0.011** |  |
| ≥ 30 kg/m^2^ | 1.04 (0.91,1.09) | 0.913 |  |  | 1.00 (0.95,1.04) | 0.981 |  |  | 0.96 (0.88,1.04) | 0.361 |  |
| **Physical activity** |  |  | 0.235 |  |  |  | 0.970 |  |  |  | 0.908 |
| Suffcient | 1.07 (0.98,1.16) | 0.106 |  |  | 1.01 (0.97,1.05) | 0.503 |  |  | 0.97 (0.89,1.05) | 0.538 |  |
| Insufficient | 0.99 (0.88,1.11) | 0.918 |  |  | 1.01 (0.95,1.07) | 0.646 |  |  | 0.97 (0.87,1.08) | 0.624 |  |
| **HDI** |  |  | 0.907 |  |  |  | 0.553 |  |  |  | 0.907 |
| < 3 | 1.08 (0.98,1.18) | 0.114 |  |  | 1.01 (0.96,1.05) | 0.650 |  |  | 1.00 (0.91,1.10) | 0.897 |  |
| ≥ 4 | 1.09 (0.98,1.20) | 0.088 |  |  | 1.02 (0.97,1.03) | 0.335 |  |  | 1.00 (0.91,1.11) | 0.859 |  |
| **Sleep duration** |  |  | 0.396 |  |  |  | 0.160 |  |  |  | 0.057 |
| < 7 hours | 1.10 (0.97,1.25) | 0.108 |  |  | 1.00 (0.94,1.06) | 0.879 |  |  | 1.04 (0.92,1.18) | 0.478 |  |
| 7-8 hours | 1.08 (0.95,1.22) | 0.198 |  |  | 1.03 (0.97,1.09) | 0.303 |  |  | 1.03 (0.92,1.17) | 0.537 |  |
| ≥ 9 hours | 1.06 (0.95,1.18) | 0.261 |  |  | 1.00 (0.95,1.06) | 0.784 |  |  | 0.94 (0.84,1.05) | 0.283 |  |
| **Dietary supplementation** |  |  | 0.476 |  |  |  | 0.242 |  |  |  | 0.298 |
| No | 1.06 (0.96,1.17) | 0.210 |  |  | 1.00 (0.95,1.04) | 0.901 |  |  | 0.97 (0.88,1.07) | 0.654 |  |
| Yes | 1.10 (1.00, 1.22) | 0.046 |  |  | 1.03 (0.98,1.08) | 0.208 |  |  | 1.03 (0.93,1.13) | 0.923 |  |
| **medication use** |  |  | 0.789 |  |  |  | 0.689 |  |  |  | 0.691 |
| No | 1.11 (1.00, 1.22) | 0.037 |  |  | 1.01 (0.96,1.06) | 0.517 |  |  | 1.03 (0.93,1.13) | 0.506 |  |
| Yes | 1.06 (0.96, 1.17) | 0.235 |  |  | 1.01 (0.97,1.06) | 0.462 |  |  | 0.98 (0.89,1.07) | 0.691 |  |

All *P* values were adjusted by false discovery rate method. Bold indicates simultaneous significance of the subgroup test and the heterogeneity test.

DWH, domestic water hardness; MASLD, Metabolic dysfunction-associated steatotic liver disease; Ca, calcium; Mg, magnesium; CaCO_3_, calcium carbonate; PM_2.5_, particulate matter with an aerodynamic diameter ≤ 2.5 μm; HR, hazard ratio; CI, confidence interval.

# Table S4 Sensitivity analysis for associations between DWH and incident MALSD risk

|  | **Ca** | | |  | **Mg** | | |  | **CaCO_3_** | | |
| --- | --- | --- | --- | --- | --- | --- | --- | --- | --- | --- | --- |
|  | **HR (95% CI)** | ***P*** | **E-value** |  | **HR (95% CI)** | ***P*** | **E-value** |  | **HR (95% CI)** | ***P*** | **E-value** |
| **Manuscript** | 1.08 (1.01,1.16) | 0.019 | 1.395 |  | 1.04 (0.99,1.09) | 0.088 | 1.254 |  | 1.00 (0.93,1.07) | 0.848 | 1.088 |
| **PM_2.5_** | 1.07 (1.00,1.15) | 0.032 | 1.367 |  | 1.00 (0.94,1.07) | 0.824 | 1.099 |  | 1.00 (0.94,1.07) | 0.824 | 1.096 |
| **Traffic noise** | 1.07(1.00,1.15) | 0.039 | 1.363 |  | 1.00 (0.97,1.03) | 0.682 | 1.085 |  | 0.99 (0.93,1.07) | 0.987 | 1.023 |
| **Green area** | 1.08 (1.01,1.15) | 0.022 | 1.381 |  | 1.01 (0.98,1.04) | 0.391 | 1.127 |  | 1.00 (0.93,1.03) | 0.843 | 1.091 |
| **Urban or rural** | 1.08 (1.00,1.16) | 0.019 | 1.395 |  | 1.00 (0.98,1.04) | 0.515 | 1.109 |  | 1.01 (0.94,1.08) | 0.755 | 1.116 |
| **Water intake** | 1.07 (1.00,1.15) | 0.038 | 1.364 |  | 1.00 (0.97,1.03) | 0.608 | 1.096 |  | 0.99 (0.93,1.06) | 0.969 | 1.037 |
| **Survey year** | 1.08 (1.00,1.16) | 0.026 | 1.380 |  | 1.00 (0.97,1.03) | 0.619 | 1.094 |  | 1.00 (0.93,1.07) | 0.920 | 1.062 |
| **Extended Cohort** | 1.06 (1.00,1.12) | 0.036 | 1.132 |  | 1.02 (1.00,1.04) | 0.034 | 1.177 |  | 0.99 (0.94,1.04) | 0.784 | 1.095 |
| **Exclusion 1*** | 1.07 (1.00,1.15) | 0.039 | 1.368 |  | 1.00 (0.97,1.03) | 0.845 | 1.058 |  | 0.99 (0.92,1.06) | 0.903 | 1.070 |
| **Exclusion 2†** | 1.08 (1.01,1.16) | 0.019 | 1.395 |  | 1.00 (0.97,1.03) | 0.628 | 1.093 |  | 1.00 (0.93,1.07) | 0.848 | 1.088 |

***** Excluding participants diagnosed within two years of baseline.

**†** Excluding participants who had lived in the area for less than 10 years.

All models were adjusted for age, sex , location, education, TDI, ethnicity, smoking status, alcohol intake, physical activity, sleep duration, BMI, and HDI, dietary supplementation and use of medications for metabolic disease.

DWH, domestic water hardness; MASLD, Metabolic dysfunction-associated steatotic liver disease; Ca, calcium; Mg, magnesium; CaCO_3_, calcium carbonate; PM_2.5_, particulate matter with an aerodynamic diameter ≤ 2.5 μm; HR, hazard ratio; CI, confidence interval.

# Table S5 Mediation analyses for identified metabolomic biomarkers in associations between Ca and incident MASLD risk

|  | TE | |  | | NDE | |  | NIE | | PM |
| --- | --- | --- | --- | --- | --- | --- | --- | --- | --- | --- |
|  | HR (95% CI) | *P* |  | HR (95% CI) | | *P* |  | HR (95% CI) | *P* |  |
| Gln | 1.088 (1.016,1.177) | 0.03 |  | 1.069 (0.997,1.154) | | 0.08 |  | 1.018 (1.013,1.022) | < 0.001 | 21.42% |
| Lactate | 1.088 (1.019,1.168) | 0.03 |  | 1.081 (1.011,1.161) | | 0.04 |  | 1.006 (1.001,1.011) | 0.03 | 7.53% |
| Ala | 1.087 (1.005,1.153) | 0.04 |  | 1.082 (0.999,1.147) | | 0.07 |  | 1.004 (1.003,1.006) | < 0.001 | 5.38% |

All models were adjusted for age, sex , location, education, TDI, ethnicity, smoking status, alcohol intake, physical activity, sleep duration, BMI, and HDI, dietary supplementation and use of medications for metabolic disease.

MASLD, metabolic dysfunction-associated steatotic liver disease; Ca, calcium; TE, total effect; NDE, natural direct effect; NIE, natural indirect effect; HR, hazard ratio; CI, confidence interval;

Gln, Glutamine; Ala,Alanine.

**Table S6 Mediation analyses for other metabolomic biomarkers in associations between Ca and incident MALSD risk**

| Mediator | TE | | NDE | | NIE | | PM |
| --- | --- | --- | --- | --- | --- | --- | --- |
|  | HR(95% CI) | *P* | HR(95% CI) | *P* | HR(95% CI) | *P* |  |
| bOHbutyrate | 1.086 (1.009, 1.172) | 0.035 | 1.086 (1.009, 1.172) | 0.035 | 1.000 (1.000, 1.001) | 0.686 | 0.2% |
| Acetate | 1.086 (1.003, 1.161) | 0.062 | 1.086 (1.003, 1.161) | 0.062 | 1.000 (1.000, 1.000) | 0.704 | -0.1% |
| Acetoacetate | 1.086 (1.014, 1.157) | 0.035 | 1.086 (1.014, 1.158) | 0.035 | 1.000 (0.999, 1.000) | 0.407 | -0.5% |
| Acetone | 1.086 (1.008, 1.167) | 0.042 | 1.085 (1.009, 1.163) | 0.035 | 1.001 (0.996, 1.004) | 0.810 | 1.0% |
| Albumin | 1.086 (1.013, 1.168) | 0.035 | 1.086 (1.014, 1.168) | 0.035 | 1.000 (0.999, 1.001) | 0.947 | 0.0% |
| ApoA1 | 1.086 (1.012, 1.154) | 0.042 | 1.087 (1.013, 1.155) | 0.042 | 0.999 (0.998, 0.999) | <0.001 | -1.6% |
| ApoB | 1.087 (1.007, 1.152) | 0.052 | 1.086 (1.006, 1.152) | 0.052 | 1.000 (1.000, 1.001) | 0.398 | 0.4% |
| HDL_size | 1.084 (1.017, 1.164) | 0.042 | 1.099 (1.032, 1.178) | 0.035 | 0.987 (0.984, 0.990) | <0.001 | -17.1% |
| LDL_size | 1.089 (1.021, 1.169) | <0.001 | 1.091 (1.021, 1.170) | <0.001 | 0.999 (0.997, 1.000) | 0.094 | -1.7% |
| VLDL_size | 1.087 (1.016, 1.169) | 0.042 | 1.098 (1.026, 1.179) | 0.027 | 0.991 (0.988, 0.992) | <0.001 | -11.9% |
| XXL_VLDL_C | 1.088 (1.002, 1.170) | 0.062 | 1.094 (1.008, 1.176) | 0.042 | 0.995 (0.993, 0.997) | <0.001 | -5.9% |
| IDL_C | 1.088 (1.013, 1.153) | 0.035 | 1.089 (1.014, 1.153) | 0.035 | 0.999 (0.998, 1.000) | 0.161 | -1.1% |
| L_HDL_C | 1.083 (1.006, 1.156) | 0.042 | 1.103 (1.025, 1.175) | 0.027 | 0.982 (0.978, 0.986) | <0.001 | -23.9% |
| L_LDL_C | 1.088 (1.015, 1.154) | 0.042 | 1.086 (1.014, 1.152) | 0.042 | 1.001 (1.000, 1.002) | 0.027 | 1.7% |
| L_VLDL_C | 1.086 (1.006, 1.168) | 0.042 | 1.094 (1.012, 1.178) | 0.027 | 0.993 (0.991, 0.995) | <0.001 | -8.5% |
| M_HDL_C | 1.086 (1.005, 1.178) | 0.052 | 1.088 (1.007, 1.181) | 0.042 | 0.998 (0.996, 0.999) | <0.001 | -2.4% |
| M_LDL_C | 1.087 (0.997, 1.167) | 0.072 | 1.086 (0.996, 1.167) | 0.072 | 1.001 (1.000, 1.002) | 0.282 | 0.7% |
| M_VLDL_C | 1.087 (1.008, 1.167) | 0.042 | 1.086 (1.007, 1.166) | 0.052 | 1.001 (1.000, 1.002) | <0.001 | 1.1% |
| S_HDL_C | 1.086 (1.017, 1.163) | 0.042 | 1.085 (1.016, 1.163) | 0.042 | 1.001 (1.000, 1.003) | 0.127 | 1.5% |
| S_LDL_C | 1.087 (1.008, 1.166) | 0.035 | 1.086 (1.009, 1.166) | 0.035 | 1.001 (1.000, 1.002) | 0.052 | 1.0% |
| S_VLDL_C | 1.086 (0.999, 1.171) | 0.072 | 1.087 (1.001, 1.172) | 0.062 | 0.999 (0.997, 0.999) | <0.001 | -1.9% |
| XL_HDL_C | 1.084 (1.020, 1.165) | 0.035 | 1.104 (1.040, 1.185) | <0.001 | 0.982 (0.977, 0.985) | <0.001 | -24.2% |
| XL_VLDL_C | 1.086 (1.019, 1.171) | 0.027 | 1.094 (1.027, 1.180) | 0.027 | 0.993 (0.991, 0.995) | <0.001 | -8.4% |
| XS_VLDL_C | 1.087 (1.024, 1.174) | 0.035 | 1.086 (1.024, 1.174) | 0.035 | 1.000 (1.000, 1.000) | 1.000 | 0.0% |
| XXL_VLDL_CE | 1.088 (1.005, 1.170) | 0.042 | 1.094 (1.010, 1.175) | 0.042 | 0.995 (0.993, 0.997) | <0.001 | -6.4% |
| HDL_CE | 1.085 (1.014, 1.162) | 0.035 | 1.094 (1.023, 1.175) | 0.027 | 0.992 (0.989, 0.995) | <0.001 | -10.7% |
| IDL_CE | 1.088 (1.015, 1.167) | 0.035 | 1.089 (1.016, 1.168) | 0.035 | 0.999 (0.998, 1.000) | 0.282 | -0.8% |
| LDL_CE | 1.087 (1.010, 1.167) | 0.035 | 1.086 (1.009, 1.166) | 0.035 | 1.001 (1.000, 1.002) | 0.027 | 1.4% |
| L_HDL_CE | 1.083 (1.006, 1.155) | 0.062 | 1.104 (1.027, 1.176) | 0.027 | 0.981 (0.977, 0.985) | <0.001 | -25.4% |
| L_LDL_CE | 1.088 (1.008, 1.174) | 0.052 | 1.086 (1.006, 1.173) | 0.062 | 1.002 (1.001, 1.002) | <0.001 | 1.9% |
| L_VLDL_CE | 1.086 (1.010, 1.159) | 0.027 | 1.091 (1.016, 1.166) | 0.027 | 0.995 (0.993, 0.997) | <0.001 | -6.0% |
| M_HDL_CE | 1.086 (1.001, 1.164) | 0.062 | 1.088 (1.004, 1.167) | 0.062 | 0.998 (0.997, 0.999) | <0.001 | -2.7% |
| M_LDL_CE | 1.086 (1.000, 1.180) | 0.062 | 1.087 (1.000, 1.181) | 0.072 | 1.000 (0.998, 1.001) | 0.572 | -0.6% |
| M_VLDL_CE | 1.089 (1.013, 1.154) | 0.035 | 1.089 (1.013, 1.153) | 0.035 | 1.000 (0.999, 1.001) | 0.969 | 0.0% |
| S_HDL_CE | 1.087 (1.009, 1.174) | 0.052 | 1.084 (1.006, 1.173) | 0.052 | 1.002 (1.001, 1.004) | <0.001 | 2.7% |
| S_LDL_CE | 1.086 (1.010, 1.164) | 0.027 | 1.086 (1.010, 1.164) | 0.027 | 1.000 (0.999, 1.001) | 0.864 | -0.2% |
| S_VLDL_CE | 1.085 (1.012, 1.174) | 0.042 | 1.088 (1.013, 1.177) | 0.027 | 0.998 (0.997, 0.999) | <0.001 | -2.7% |
| VLDL_CE | 1.086 (1.008, 1.166) | 0.042 | 1.087 (1.010, 1.167) | 0.042 | 0.999 (0.998, 1.000) | <0.001 | -1.1% |
| XL_HDL_CE | 1.084 (1.008, 1.160) | 0.062 | 1.105 (1.027, 1.181) | <0.001 | 0.981 (0.976, 0.984) | <0.001 | -25.5% |
| XL_VLDL_CE | 1.086 (1.000, 1.159) | 0.062 | 1.092 (1.006, 1.166) | 0.052 | 0.994 (0.992, 0.996) | <0.001 | -7.0% |
| XS_VLDL_CE | 1.087 (1.011, 1.163) | 0.035 | 1.087 (1.012, 1.163) | 0.035 | 1.000 (0.999, 1.000) | 0.369 | -0.3% |
| Citrate | 1.086 (1.018, 1.175) | 0.027 | 1.086 (1.018, 1.175) | 0.027 | 1.000 (1.000, 1.000) | 0.827 | -0.0% |
| Clinical_LDL_C | 1.088 (1.021, 1.155) | 0.035 | 1.087 (1.019, 1.153) | 0.035 | 1.001 (1.000, 1.002) | <0.001 | 1.6% |
| XXL_VLDL_P | 1.089 (1.015, 1.172) | 0.035 | 1.094 (1.020, 1.178) | 0.035 | 0.995 (0.993, 0.996) | <0.001 | -6.3% |
| HDL_P | 1.086 (1.021, 1.169) | <0.001 | 1.086 (1.021, 1.169) | <0.001 | 1.000 (0.999, 1.001) | 0.845 | 0.1% |
| IDL_P | 1.087 (1.021, 1.166) | 0.027 | 1.087 (1.020, 1.167) | 0.027 | 1.000 (0.999, 1.000) | 0.205 | -0.5% |
| LDL_P | 1.087 (1.007, 1.164) | 0.027 | 1.086 (1.007, 1.164) | 0.035 | 1.001 (1.000, 1.001) | 0.116 | 0.7% |
| L_HDL_P | 1.083 (1.013, 1.164) | <0.001 | 1.100 (1.028, 1.183) | <0.001 | 0.985 (0.981, 0.989) | <0.001 | -19.8% |
| L_LDL_P | 1.087 (1.006, 1.156) | 0.042 | 1.086 (1.005, 1.155) | 0.042 | 1.001 (1.000, 1.002) | 0.027 | 1.2% |
| L_VLDL_P | 1.087 (1.015, 1.162) | <0.001 | 1.097 (1.025, 1.172) | <0.001 | 0.991 (0.989, 0.993) | <0.001 | -10.8% |
| M_HDL_P | 1.086 (1.016, 1.158) | 0.027 | 1.087 (1.016, 1.159) | 0.027 | 0.999 (0.998, 1.000) | <0.001 | -1.2% |
| M_LDL_P | 1.086 (1.011, 1.153) | 0.035 | 1.086 (1.012, 1.153) | 0.035 | 1.000 (0.999, 1.001) | 0.578 | -0.4% |
| M_VLDL_P | 1.086 (1.007, 1.160) | 0.042 | 1.088 (1.008, 1.163) | 0.042 | 0.998 (0.997, 0.999) | <0.001 | -2.4% |
| S_HDL_P | 1.086 (1.011, 1.157) | 0.042 | 1.086 (1.011, 1.158) | 0.042 | 1.000 (0.999, 1.002) | 0.637 | 0.2% |
| S_LDL_P | 1.086 (1.009, 1.168) | 0.035 | 1.086 (1.009, 1.168) | 0.035 | 1.000 (0.999, 1.000) | 0.389 | -0.4% |
| S_VLDL_P | 1.086 (1.018, 1.182) | 0.027 | 1.093 (1.024, 1.191) | <0.001 | 0.994 (0.992, 0.995) | <0.001 | -7.9% |
| VLDL_P | 1.086 (1.014, 1.171) | 0.042 | 1.090 (1.017, 1.176) | 0.035 | 0.996 (0.994, 0.997) | <0.001 | -5.4% |
| XL_HDL_P | 1.084 (1.013, 1.165) | <0.001 | 1.099 (1.027, 1.182) | <0.001 | 0.986 (0.983, 0.989) | <0.001 | -17.8% |
| XL_VLDL_P | 1.088 (1.015, 1.163) | 0.027 | 1.098 (1.024, 1.174) | 0.027 | 0.991 (0.990, 0.993) | <0.001 | -10.8% |
| XS_VLDL_P | 1.086 (1.010, 1.179) | 0.035 | 1.087 (1.011, 1.180) | 0.035 | 0.999 (0.998, 1.000) | <0.001 | -1.1% |
| Creatinine | 1.086 (1.023, 1.164) | 0.027 | 1.087 (1.024, 1.165) | 0.027 | 0.999 (0.998, 1.000) | 0.116 | -1.1% |
| Unsaturation | 1.093 (1.020, 1.171) | 0.035 | 1.110 (1.036, 1.187) | <0.001 | 0.985 (0.982, 0.988) | <0.001 | -18.5% |
| DHA | 1.087 (1.003, 1.159) | 0.035 | 1.090 (1.007, 1.161) | 0.035 | 0.998 (0.996, 0.999) | <0.001 | -2.9% |
| XXL_VLDL_FC | 1.089 (1.017, 1.175) | 0.035 | 1.093 (1.021, 1.180) | 0.035 | 0.996 (0.994, 0.998) | <0.001 | -5.0% |
| HDL_FC | 1.085 (1.016, 1.164) | 0.035 | 1.090 (1.022, 1.168) | <0.001 | 0.995 (0.993, 0.997) | <0.001 | -6.0% |
| IDL_FC | 1.088 (1.010, 1.160) | 0.052 | 1.090 (1.011, 1.161) | 0.052 | 0.998 (0.997, 1.000) | 0.027 | -1.9% |
| LDL_FC | 1.088 (1.015, 1.160) | 0.042 | 1.087 (1.013, 1.157) | 0.042 | 1.001 (1.000, 1.002) | 0.261 | 1.1% |
| L_HDL_FC | 1.084 (1.019, 1.172) | 0.035 | 1.099 (1.034, 1.190) | 0.035 | 0.986 (0.982, 0.989) | <0.001 | -18.0% |
| L_LDL_FC | 1.088 (1.014, 1.160) | 0.027 | 1.088 (1.013, 1.161) | 0.027 | 1.000 (0.999, 1.001) | 0.900 | 0.1% |
| L_VLDL_FC | 1.087 (1.018, 1.167) | <0.001 | 1.096 (1.026, 1.175) | <0.001 | 0.992 (0.990, 0.994) | <0.001 | -10.5% |
| M_HDL_FC | 1.086 (1.010, 1.157) | 0.035 | 1.087 (1.011, 1.158) | 0.035 | 0.999 (0.998, 1.000) | <0.001 | -1.5% |
| M_LDL_FC | 1.088 (1.024, 1.175) | <0.001 | 1.086 (1.023, 1.173) | <0.001 | 1.002 (1.001, 1.003) | <0.001 | 2.3% |
| M_VLDL_FC | 1.086 (1.015, 1.165) | <0.001 | 1.086 (1.016, 1.166) | <0.001 | 1.000 (0.999, 1.001) | 0.509 | -0.4% |
| S_HDL_FC | 1.086 (1.012, 1.166) | 0.027 | 1.088 (1.014, 1.168) | 0.027 | 0.999 (0.997, 1.000) | 0.035 | -1.8% |
| S_LDL_FC | 1.089 (1.023, 1.164) | 0.035 | 1.086 (1.022, 1.162) | 0.035 | 1.002 (1.001, 1.003) | <0.001 | 2.6% |
| S_VLDL_FC | 1.086 (1.007, 1.160) | 0.052 | 1.086 (1.007, 1.161) | 0.052 | 1.000 (0.999, 1.001) | 0.509 | -0.4% |
| VLDL_FC | 1.086 (1.022, 1.176) | 0.027 | 1.090 (1.026, 1.180) | <0.001 | 0.996 (0.994, 0.997) | <0.001 | -5.3% |
| XL_HDL_FC | 1.085 (1.020, 1.171) | 0.027 | 1.100 (1.033, 1.187) | <0.001 | 0.987 (0.983, 0.990) | <0.001 | -17.0% |
| XL_VLDL_FC | 1.087 (1.013, 1.174) | 0.035 | 1.095 (1.022, 1.184) | 0.035 | 0.993 (0.991, 0.995) | <0.001 | -8.8% |
| XS_VLDL_FC | 1.086 (1.008, 1.178) | 0.052 | 1.087 (1.009, 1.179) | 0.052 | 0.999 (0.999, 1.000) | 0.027 | -0.8% |
| Glucose | 1.084 (1.010, 1.168) | 0.052 | 1.094 (1.021, 1.177) | 0.035 | 0.991 (0.989, 0.993) | <0.001 | -11.6% |
| Gln | 1.088 (1.016, 1.177) | 0.035 | 1.069 (0.997, 1.154) | 0.083 | 1.018 (1.013, 1.022) | <0.001 | 21.4% |
| Gly | 1.086 (1.011, 1.165) | 0.035 | 1.087 (1.011, 1.165) | 0.035 | 1.000 (0.998, 1.001) | 0.500 | -0.5% |
| GlycA | 1.085 (1.020, 1.178) | 0.027 | 1.095 (1.029, 1.190) | 0.027 | 0.990 (0.988, 0.993) | <0.001 | -12.4% |
| HDL_C | 1.085 (1.009, 1.169) | 0.027 | 1.093 (1.018, 1.178) | 0.027 | 0.992 (0.989, 0.995) | <0.001 | -9.9% |
| His | 1.086 (1.011, 1.157) | 0.027 | 1.087 (1.011, 1.158) | 0.027 | 1.000 (0.998, 1.001) | 0.742 | -0.5% |
| Ile | 1.086 (1.010, 1.165) | 0.052 | 1.089 (1.013, 1.167) | 0.052 | 0.998 (0.997, 0.999) | <0.001 | -2.6% |
| LDL_C | 1.087 (1.022, 1.160) | 0.035 | 1.086 (1.020, 1.159) | 0.035 | 1.001 (1.001, 1.002) | <0.001 | 1.7% |
| Lactate | 1.088 (1.019, 1.168) | 0.035 | 1.081 (1.011, 1.161) | 0.042 | 1.006 (1.001, 1.011) | 0.035 | 7.5% |
| Leu | 1.087 (1.005, 1.172) | 0.035 | 1.090 (1.008, 1.176) | 0.027 | 0.997 (0.995, 0.998) | <0.001 | -4.0% |
| LA | 1.087 (1.010, 1.172) | 0.052 | 1.087 (1.010, 1.173) | 0.052 | 1.000 (0.999, 1.000) | 0.094 | -0.5% |
| MUFA | 1.088 (1.015, 1.174) | 0.042 | 1.098 (1.024, 1.184) | 0.027 | 0.991 (0.989, 0.993) | <0.001 | -11.1% |
| Omega_3 | 1.086 (1.009, 1.171) | 0.035 | 1.085 (1.009, 1.170) | 0.035 | 1.001 (1.000, 1.001) | 0.094 | 0.7% |
| Omega_6 | 1.087 (1.025, 1.163) | 0.042 | 1.086 (1.025, 1.163) | 0.042 | 1.000 (1.000, 1.001) | 0.150 | 0.4% |
| Phe | 1.087 (1.004, 1.170) | 0.042 | 1.090 (1.007, 1.173) | 0.042 | 0.997 (0.996, 0.998) | <0.001 | -3.9% |
| Phosphatidylc | 1.086 (0.996, 1.158) | 0.083 | 1.086 (0.996, 1.158) | 0.083 | 1.000 (1.000, 1.000) | 0.919 | -0.0% |
| Phosphoglyc | 1.086 (1.013, 1.165) | 0.042 | 1.087 (1.014, 1.166) | 0.042 | 1.000 (0.999, 1.000) | 0.183 | -0.5% |
| XXL_VLDL_PL | 1.089 (1.018, 1.159) | 0.035 | 1.094 (1.023, 1.164) | 0.035 | 0.996 (0.993, 0.997) | <0.001 | -5.5% |
| HDL_PL | 1.086 (1.016, 1.158) | 0.035 | 1.087 (1.017, 1.159) | 0.035 | 0.999 (0.998, 1.000) | <0.001 | -1.5% |
| IDL_PL | 1.087 (1.014, 1.162) | 0.042 | 1.088 (1.014, 1.162) | 0.042 | 1.000 (0.999, 1.001) | 0.770 | -0.2% |
| LDL_PL | 1.087 (1.002, 1.157) | 0.052 | 1.086 (1.001, 1.155) | 0.062 | 1.001 (1.000, 1.002) | <0.001 | 1.5% |
| L_HDL_PL | 1.084 (1.009, 1.154) | 0.052 | 1.095 (1.019, 1.166) | 0.042 | 0.990 (0.987, 0.992) | <0.001 | -12.9% |
| L_LDL_PL | 1.088 (1.009, 1.170) | 0.052 | 1.086 (1.008, 1.169) | 0.052 | 1.001 (1.000, 1.002) | <0.001 | 1.4% |
| L_VLDL_PL | 1.087 (1.019, 1.190) | <0.001 | 1.097 (1.028, 1.202) | <0.001 | 0.991 (0.989, 0.993) | <0.001 | -11.3% |
| M_HDL_PL | 1.086 (1.014, 1.163) | 0.052 | 1.086 (1.014, 1.163) | 0.052 | 1.000 (1.000, 1.000) | 0.963 | 0.0% |
| M_LDL_PL | 1.087 (1.000, 1.160) | 0.062 | 1.086 (1.000, 1.160) | 0.072 | 1.000 (0.999, 1.002) | 0.449 | 0.6% |
| M_VLDL_PL | 1.086 (1.000, 1.169) | 0.062 | 1.087 (1.002, 1.171) | 0.052 | 0.998 (0.997, 0.999) | 0.027 | -2.0% |
| S_HDL_PL | 1.087 (1.013, 1.157) | 0.035 | 1.090 (1.017, 1.160) | 0.035 | 0.996 (0.994, 0.998) | <0.001 | -4.5% |
| S_LDL_PL | 1.087 (1.024, 1.169) | 0.035 | 1.086 (1.024, 1.168) | 0.035 | 1.001 (1.000, 1.002) | 0.042 | 1.2% |
| S_VLDL_PL | 1.086 (1.015, 1.164) | <0.001 | 1.088 (1.018, 1.168) | <0.001 | 0.998 (0.996, 0.999) | <0.001 | -3.1% |
| VLDL_PL | 1.086 (1.018, 1.174) | <0.001 | 1.092 (1.024, 1.180) | <0.001 | 0.995 (0.993, 0.996) | <0.001 | -6.9% |
| XL_HDL_PL | 1.084 (1.015, 1.180) | 0.042 | 1.099 (1.030, 1.196) | 0.035 | 0.986 (0.982, 0.989) | <0.001 | -18.7% |
| XL_VLDL_PL | 1.088 (1.016, 1.178) | 0.042 | 1.096 (1.023, 1.188) | <0.001 | 0.992 (0.990, 0.995) | <0.001 | -9.5% |
| XS_VLDL_PL | 1.086 (1.015, 1.149) | 0.035 | 1.088 (1.017, 1.151) | 0.035 | 0.998 (0.997, 0.999) | <0.001 | -2.2% |
| PUFA | 1.087 (1.017, 1.159) | 0.027 | 1.086 (1.017, 1.158) | 0.027 | 1.000 (1.000, 1.000) | 0.713 | 0.1% |
| Pyruvate | 1.084 (1.011, 1.158) | <0.001 | 1.090 (1.019, 1.165) | <0.001 | 0.995 (0.989, 1.002) | 0.172 | -6.7% |
| Remnant_C | 1.086 (1.012, 1.163) | <0.001 | 1.086 (1.012, 1.163) | <0.001 | 1.000 (1.000, 1.001) | 0.578 | 0.2% |
| SFA | 1.088 (1.014, 1.167) | 0.027 | 1.095 (1.022, 1.175) | <0.001 | 0.993 (0.991, 0.995) | <0.001 | -8.7% |
| Ala | 1.087 (1.005, 1.153) | 0.042 | 1.082 (0.999, 1.147) | 0.072 | 1.004 (1.003, 1.006) | <0.001 | 5.4% |
| Sphingomyelins | 1.087 (1.015, 1.181) | 0.035 | 1.085 (1.012, 1.180) | 0.035 | 1.002 (1.001, 1.003) | <0.001 | 2.4% |
| Total_C | 1.087 (1.017, 1.166) | 0.042 | 1.087 (1.017, 1.166) | 0.042 | 1.000 (0.999, 1.001) | 0.358 | 0.5% |
| non_HDL_C | 1.087 (1.023, 1.171) | <0.001 | 1.086 (1.023, 1.171) | <0.001 | 1.001 (1.000, 1.002) | 0.027 | 0.9% |
| Cholines | 1.086 (0.998, 1.176) | 0.072 | 1.086 (0.998, 1.176) | 0.072 | 1.000 (1.000, 1.001) | 0.449 | 0.1% |
| Total_BCAA | 1.086 (1.016, 1.169) | <0.001 | 1.090 (1.019, 1.173) | <0.001 | 0.996 (0.995, 0.998) | <0.001 | -4.5% |
| Total_P | 1.086 (1.010, 1.159) | 0.035 | 1.086 (1.010, 1.158) | 0.035 | 1.000 (1.000, 1.001) | 0.398 | 0.5% |
| Total_CE | 1.088 (1.026, 1.177) | <0.001 | 1.087 (1.026, 1.178) | <0.001 | 1.000 (0.999, 1.001) | 0.627 | 0.4% |
| Total_FA | 1.086 (1.013, 1.162) | 0.035 | 1.091 (1.018, 1.166) | 0.027 | 0.995 (0.994, 0.997) | <0.001 | -5.7% |
| Total_FC | 1.087 (1.016, 1.163) | 0.042 | 1.087 (1.016, 1.162) | 0.042 | 1.001 (1.000, 1.001) | 0.094 | 0.6% |
| XXL_VLDL_L | 1.089 (1.014, 1.167) | 0.035 | 1.094 (1.019, 1.174) | <0.001 | 0.995 (0.993, 0.997) | <0.001 | -6.0% |
| HDL_L | 1.085 (1.016, 1.166) | 0.042 | 1.088 (1.019, 1.170) | 0.035 | 0.997 (0.996, 0.999) | <0.001 | -3.5% |
| IDL_L | 1.087 (1.020, 1.169) | 0.027 | 1.088 (1.021, 1.168) | 0.027 | 1.000 (0.999, 1.001) | 0.704 | -0.3% |
| LDL_L | 1.087 (1.010, 1.166) | 0.042 | 1.086 (1.010, 1.164) | 0.042 | 1.001 (1.000, 1.002) | <0.001 | 1.3% |
| L_HDL_L | 1.084 (1.010, 1.160) | 0.035 | 1.098 (1.025, 1.177) | 0.027 | 0.987 (0.983, 0.990) | <0.001 | -17.3% |
| L_LDL_L | 1.087 (1.005, 1.163) | 0.035 | 1.086 (1.004, 1.161) | 0.035 | 1.001 (1.000, 1.002) | <0.001 | 1.4% |
| L_VLDL_L | 1.087 (1.004, 1.172) | 0.042 | 1.097 (1.013, 1.181) | 0.035 | 0.991 (0.989, 0.993) | <0.001 | -11.0% |
| Total_L | 1.086 (1.017, 1.155) | 0.027 | 1.087 (1.017, 1.156) | 0.027 | 0.999 (0.998, 1.000) | 0.083 | -0.9% |
| M_HDL_L | 1.086 (1.016, 1.161) | 0.035 | 1.086 (1.017, 1.161) | 0.035 | 1.000 (0.999, 1.000) | 0.358 | -0.3% |
| M_LDL_L | 1.086 (1.013, 1.170) | 0.035 | 1.086 (1.013, 1.170) | 0.035 | 1.000 (0.999, 1.001) | 0.977 | 0.1% |
| M_VLDL_L | 1.086 (1.010, 1.172) | 0.035 | 1.090 (1.014, 1.176) | 0.035 | 0.996 (0.995, 0.998) | <0.001 | -4.7% |
| S_HDL_L | 1.086 (1.019, 1.160) | 0.042 | 1.090 (1.022, 1.163) | 0.042 | 0.997 (0.995, 0.998) | <0.001 | -4.0% |
| S_LDL_L | 1.086 (1.021, 1.161) | <0.001 | 1.086 (1.020, 1.161) | <0.001 | 1.000 (1.000, 1.001) | 0.597 | 0.4% |
| S_VLDL_L | 1.086 (1.013, 1.165) | 0.052 | 1.092 (1.017, 1.172) | 0.042 | 0.994 (0.992, 0.996) | <0.001 | -7.3% |
| VLDL_L | 1.087 (1.003, 1.182) | 0.062 | 1.094 (1.009, 1.191) | 0.035 | 0.993 (0.991, 0.995) | <0.001 | -8.6% |
| XL_HDL_L | 1.084 (0.994, 1.170) | 0.072 | 1.101 (1.009, 1.190) | 0.042 | 0.985 (0.981, 0.988) | <0.001 | -20.1% |
| XL_VLDL_L | 1.088 (1.023, 1.165) | <0.001 | 1.098 (1.031, 1.175) | <0.001 | 0.991 (0.989, 0.993) | <0.001 | -10.7% |
| XS_VLDL_L | 1.086 (0.994, 1.176) | 0.083 | 1.087 (0.995, 1.177) | 0.072 | 0.999 (0.998, 1.000) | <0.001 | -1.5% |
| Total_PL | 1.086 (0.998, 1.165) | 0.072 | 1.086 (0.998, 1.165) | 0.072 | 1.000 (1.000, 1.000) | 0.984 | 0.0% |
| Total_TG | 1.088 (1.013, 1.164) | 0.027 | 1.098 (1.021, 1.173) | <0.001 | 0.991 (0.989, 0.993) | <0.001 | -10.8% |
| XXL_VLDL_TG | 1.089 (1.005, 1.174) | 0.042 | 1.094 (1.009, 1.181) | 0.035 | 0.995 (0.993, 0.997) | <0.001 | -5.9% |
| HDL_TG | 1.090 (1.010, 1.170) | 0.035 | 1.095 (1.016, 1.176) | 0.035 | 0.995 (0.993, 0.997) | <0.001 | -6.0% |
| IDL_TG | 1.088 (1.010, 1.169) | 0.052 | 1.094 (1.016, 1.177) | 0.035 | 0.994 (0.992, 0.996) | <0.001 | -7.0% |
| LDL_TG | 1.088 (1.010, 1.173) | 0.042 | 1.094 (1.015, 1.180) | 0.035 | 0.994 (0.992, 0.996) | <0.001 | -7.0% |
| L_HDL_TG | 1.088 (1.010, 1.172) | <0.001 | 1.087 (1.010, 1.170) | 0.027 | 1.002 (1.001, 1.003) | <0.001 | 2.0% |
| L_LDL_TG | 1.088 (1.012, 1.161) | 0.042 | 1.094 (1.016, 1.167) | 0.027 | 0.995 (0.993, 0.996) | <0.001 | -6.6% |
| L_VLDL_TG | 1.088 (1.013, 1.180) | 0.042 | 1.098 (1.022, 1.190) | 0.035 | 0.991 (0.989, 0.993) | <0.001 | -11.3% |
| M_HDL_TG | 1.089 (1.017, 1.165) | 0.027 | 1.096 (1.024, 1.173) | 0.027 | 0.994 (0.992, 0.996) | <0.001 | -7.1% |
| M_LDL_TG | 1.088 (1.016, 1.166) | <0.001 | 1.095 (1.023, 1.173) | <0.001 | 0.994 (0.992, 0.996) | <0.001 | -7.3% |
| M_VLDL_TG | 1.087 (1.011, 1.156) | <0.001 | 1.096 (1.018, 1.166) | <0.001 | 0.992 (0.990, 0.993) | <0.001 | -10.7% |
| S_HDL_TG | 1.089 (1.009, 1.186) | 0.042 | 1.101 (1.018, 1.200) | <0.001 | 0.989 (0.986, 0.991) | <0.001 | -13.8% |
| S_LDL_TG | 1.088 (1.005, 1.179) | 0.042 | 1.095 (1.012, 1.185) | 0.035 | 0.994 (0.992, 0.996) | <0.001 | -7.1% |
| S_VLDL_TG | 1.088 (1.018, 1.158) | 0.027 | 1.098 (1.029, 1.168) | 0.027 | 0.990 (0.988, 0.992) | <0.001 | -12.4% |
| VLDL_TG | 1.088 (1.013, 1.159) | 0.027 | 1.098 (1.023, 1.169) | <0.001 | 0.991 (0.989, 0.993) | <0.001 | -10.9% |
| XL_HDL_TG | 1.089 (1.018, 1.177) | <0.001 | 1.088 (1.018, 1.178) | <0.001 | 1.000 (0.999, 1.001) | 0.991 | 0.1% |
| XL_VLDL_TG | 1.089 (1.009, 1.184) | 0.035 | 1.099 (1.018, 1.194) | <0.001 | 0.991 (0.989, 0.993) | <0.001 | -11.2% |
| XS_VLDL_TG | 1.089 (1.005, 1.176) | 0.042 | 1.097 (1.011, 1.183) | 0.042 | 0.992 (0.990, 0.994) | <0.001 | -9.4% |
| Tyr | 1.087 (1.017, 1.167) | 0.035 | 1.098 (1.028, 1.180) | 0.027 | 0.990 (0.987, 0.993) | <0.001 | -12.8% |
| VLDL_C | 1.086 (1.011, 1.172) | 0.042 | 1.088 (1.014, 1.175) | 0.042 | 0.998 (0.997, 0.999) | <0.001 | -2.7% |
| Val | 1.086 (1.008, 1.171) | 0.042 | 1.090 (1.011, 1.175) | 0.042 | 0.996 (0.994, 0.997) | <0.001 | -5.0% |

All models were adjusted for age, sex , location, education, TDI, ethnicity, smoking status, alcohol intake, physical activity, sleep duration, BMI, and HDI, dietary supplementation and use of medications for metabolic disease.

All *P* values were FDR-adjusted.
